# Mitochondria: Novel Mechanisms and Therapeutic Targets for Secondary Brain Injury After Intracerebral Hemorrhage

**DOI:** 10.3389/fnagi.2020.615451

**Published:** 2021-01-27

**Authors:** Weixiang Chen, Chao Guo, Hua Feng, Yujie Chen

**Affiliations:** ^1^Department of Neurosurgery, Southwest Hospital, Third Military Medical University (Army Medical University), Chongqing, China; ^2^State Key Laboratory of Trauma, Burn and Combined Injury, Third Military Medical University (Army Medical University), Chongqing, China; ^3^Chongqing Key Laboratory of Precision Neuromedicine and Neuroregenaration, Third Military Medical University (Army Medical University), Chongqing, China; ^4^Collaborative Innovation Center for Brain Science, Third Military Medical University (Army Medical University), Chongqing, China

**Keywords:** intracerebral hemorrhage, mitochondrial protection, secondary brain injury, mitochondrial membrane potential, stroke

## Abstract

Intracerebral hemorrhage (ICH) is a destructive form of stroke that often results in death or disability. However, the survivors usually experience sequelae of neurological impairments and psychiatric disorders, which affect their daily functionality and working capacity. The recent MISTIE III and STICH II trials have confirmed that early surgical clearance of hematomas does not improve the prognosis of survivors of ICH, so it is vital to find the intervention target of secondary brain injury (SBI) after ICH. Mitochondrial dysfunction, which may be induced by oxidative stress, neuroinflammation, and autophagy, among others, is considered to be a novel pathological mechanism of ICH. Moreover, mitochondria play an important role in promoting neuronal survival and improving neurological function after a hemorrhagic stroke. This review summarizes the mitochondrial mechanism involved in cell death, reactive oxygen species (ROS) production, inflammatory activation, blood–brain barrier (BBB) disruption, and brain edema underlying ICH. We emphasize the potential of mitochondrial protection as a potential therapeutic target for SBI after stroke and provide valuable insight into clinical strategies.

## Introduction

Hemorrhagic stroke, which is less common but far more likely to be fatal, makes up about 13% of stroke cases. Indeed, two-thirds of stroke survivors will experience moderate or severe disability (Balami and Buchan, 2012). The high incidence and mortality of intracerebral hemorrhage (ICH) are due to both primary brain injury (mainly caused by a mass effect) and secondary brain injury (SBI; mainly caused by hemoglobin degradation products), including neuronal death, oxidative stress injury, and cerebral edema (Wang et al., 2002; Cordonnier et al., 2018). Recently, the MISTIE III and STICH II trials confirmed that early surgical clearance of hematomas does not improve the prognosis of patients with ICH (Mendelow et al., 2013; Hanley et al., 2019), and the lack of evidence-based surgical treatment strategies has prompted researchers to seek effective intervention targets and therapies for secondary injuries after ICH.

The prevalence of ICH is ~120 per 100,000; 58% of patients die within 1 year, with two-thirds of the survivors being moderately or severely disabled (Balami and Buchan, 2012; Kumar et al., 2016). Furthermore, the 30-days mortality rate is ~30–55% (Feigin et al., 2015). ICH is associated with high morbidity and mortality from severe SBI, including white matter damage, neuronal death (apoptosis and necrosis), and inflammatory changes (Bobinger et al., 2018).

As the powerhouse of cells, mitochondria play a crucial role in cell energy homeostasis; thus, they are inevitably associated with the pathophysiology of ICH (Georgieva et al., 2017; Chen et al., 2020b). Mitochondrial injury in patients with ICH was first reported in 2006, suggesting that the damage is not caused by hypoxia and ischemia but rather by mitochondrial dysfunction (Kim-Han et al., 2006). Based on the knowledge gained by the greater attention paid to the development of methods for mitochondrial research, it is currently believed that the function of mitochondria involves both supplying energy for the cell and regulating cell death and inflammation (Dawson and Dawson, 2017; Gong et al., 2018). Metformin, a mitochondrion-related treatment drug, has been shown to reduce apoptosis, inflammation, oxidative stress, DNA damage, and mitochondrial damage after cerebral hemorrhage (Wang et al., 2018). Additionally, mitoquinone (MitoQ), a mitochondrial reactive oxygen-scavenging agent, improves mitochondrial function and motor function after ICH, reduces the hematoma volume, and alleviates cerebral edema (Chen et al., 2020a,b).

In this review, we discuss the latest perspectives on the role of mitochondria in cell death and survival and highlight the pathogenesis and treatment of mitochondrion-associated ICH.

## Pathophysiology of ICH

**Figure 1:**
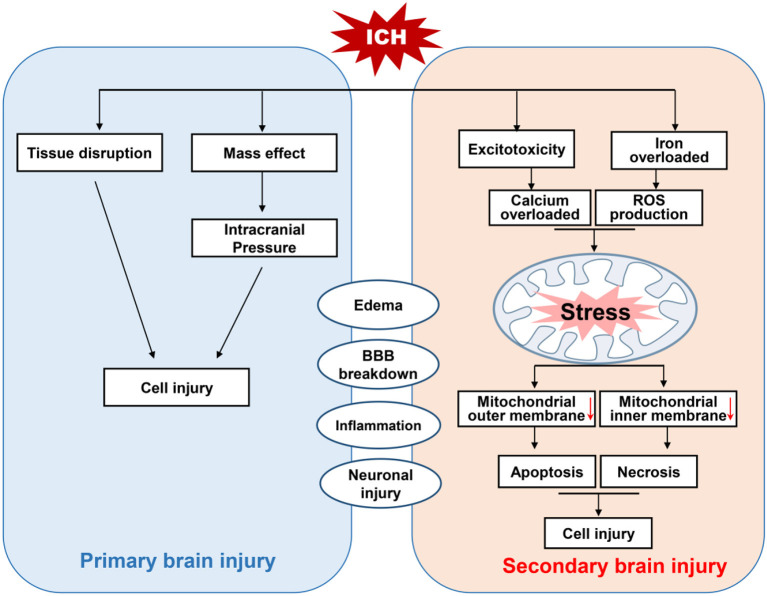
Schematic diagram of brain injury after intracerebral hemorrhage (ICH). Brain injury after ICH can be divided into primary and secondary brain injury (SBI). The primary brain injury is caused by tissue disruption and mass effect after initial bleeding. Whereas, SBI is induced by oxidative stress, the activation of TRP channels and excitotoxicity due to the accumulation of reactive oxygen species (ROS) triggered by iron overload and calcium. This excessive ROS and calcium cause mitochondrial dysfunction, and the activation of apoptotic factors leads to apoptotic and necrotic cell death.

Intracerebral hemorrhage is a subtype of stroke that is associated with high rates of mortality and disability (Feigin et al., 2015). There are two million cases of ICH worldwide each year, and those who survive often have severe neurological deficits (Kumar et al., 2016). At present, the mainstream view is that nerve injury after ICH can be divided into primary brain injury and SBI (Figure 1; Aronowski and Zhao, 2011). The former is mainly caused by mechanical disruption after initial bleeding, whereas the latter is caused by a series of mechanisms including oxidative stress, inflammation, mitochondrial dysfunction, and neuronal death (including apoptosis and necrosis; Yu et al., 2019).

Although most researchers believe that these mechanisms are related to SBI after ICH, effective interventions are still lacking (Cordonnier et al., 2018). Therefore, it is important to explore ways of promoting the recovery of nerve function by reducing secondary injury in the treatment of ICH.

## Structure and Function of Mitochondria

**Figure 2:**
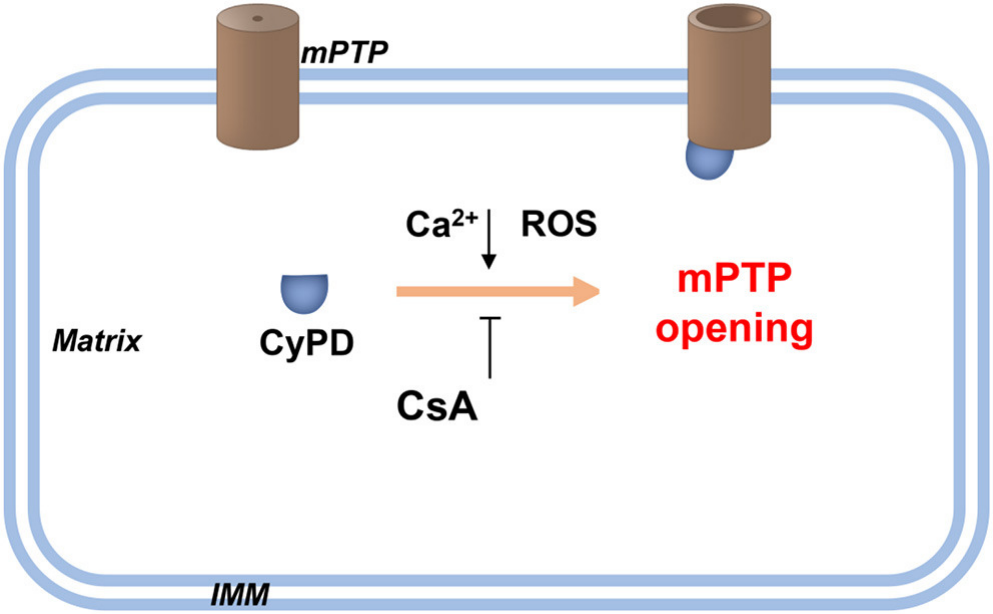
Schematic diagram of the PTP opening hypothesis. As illustrated in the figure above, an increasing level of Ca^2+^ and/or ROS in the mitochondrial matrix promotes the opening of high conductance of the PTP. This process is achieved by combining CyPD with PTP. Therefore, drugs targeting CypD, such as CsA, or genetic interventions for CyPD reduction can be applied without affecting PTP opening. Recently, Ca^2+^-induced PTP opening *via* small conductance PTP has been reported, and this process inhibits CsA. However, the same protein with different conformations may form pores of increasing size. CsA, cyclosporin A; CyPD, cyclophylin D; IMM, inner mitochondrial membrane; ROS, reactive oxygen species.

Mitochondria are ovoid or rod-shaped organelles with double-membrane structures comprising four distinct compartments: the outer membrane, the intermembrane space, the inner membrane, and the matrix (Figure 2; Giacomello et al., 2020). These organelles perform a variety of key functions in different cellular processes (Cunnane et al., 2020). The human nervous system consumes a great amount of energy, and by providing ATP through oxidative phosphorylation, mitochondria are the main source of energy for normal neuronal homeostasis and function (Area-Gomez et al., 2019).

Indeed, mitochondria are pivotal regulators of cell survival, and their crucial role in cell survival is mainly reflected in three aspects (Bock and Tait, 2020). Mitochondria are the hubs of cellular calcium signaling, and they provide an important mechanism for regulating calcium concentration during signal transduction, which is particularly important for excitable cells, such as neurons (Giorgi et al., 2018). The enzyme complexes located in the mitochondrial inner membrane are essential for maintaining cellular energy requirements and metabolic homeostasis (Fricker et al., 2018). The survival of neuronal cells depends largely on the integrity and function of mitochondria. Therefore, mitochondrial defects have severe destructive effects on the central nervous system (CNS; Zsurka and Kunz, 2015).

## Mitochondrial Quality Control Systems and ICH

Mitochondria control almost every aspect of cellular function, including managing REDOX status, modulating Ca^2+^ homeostasis, generating ATP, and regulating responses to cellular and environmental stresses (Liu et al., 2018). The function of mitochondria is also important for the health of neurons, and the activities of axons and dendritic neuron fibers are highly dependent on mitochondria, which are highly dynamic and exhibit an activity-induced interval distribution (Eisner et al., 2018; Bock and Tait, 2020). For example, mitochondrial transport to the remote synapse provides sufficient energy for local synaptic activity in the maintenance of ion channels, transporters, and synaptic transmission (Licznerski et al., 2020).

Moreover, abundant evidence supports the importance of maintaining normal clearance of damaged mitochondria for neuron survival after ICH (Huang and Jiang, 2019; Li et al., 2020). Mitochondrial dysfunction in SBI after ICH has been well-described, and mitochondrial quality and transport have a key function in the protection of neuronal injury. In fact, maintaining mitochondrial integrity and removing damaged mitochondria are important ways to prevent extensive mitochondrial dysfunction, oxidative stress, and cell death caused by hemorrhagic injury (Huang and Jiang, 2019). Therefore, mitochondrial dynamics and mitophagy are vital for maintaining cellular homeostasis and function after ICH. Here, we systematically review the critical roles of mitochondrial integrity and function in neuronal survival after ICH.

### Mitochondrial ROS and ICH

Reactive oxygen species (ROS) are the causes of SBI after ICH (Duan et al., 2016; Qu et al., 2016). Excessive accumulation of ROS can lead to macromolecular damage, cellular signal transduction disorder, cell death, and tissue damage (Forrester et al., 2018).

After ICH, hematoma and perihematomal regions are rich with RBC lysis products, especially hemin. In addition, intracellular hemin is degraded into Fe^2+^. Fenton reaction caused by Fe^2+^ can generate a hydroxyl radical, which is the most reactive of all oxygen radicals, and leads to oxidative stress (Zille et al., 2017; Bertero and Maack, 2018). The initial bleed leads to an influx of glutamate from the bloodstream, and excessive glutamate is one of the most important damaging factors in the nervous system (Cheng et al., 2016). Excessive glutamate in the brain parenchyma can induce Ca^2+^ overload, which leads to membrane depolarization and ROS release (Joshi et al., 2015). Activation of inflammatory cells also contributes to the pathogenesis of brain injury in ICH, and granulocytes can be a source of ROS after ICH. They can cause the release of ROS *via* nicotine adenine dinucleotide phosphate (NADPH) oxidase and myeloperoxidase (Zia et al., 2009).

**Figure 3:**
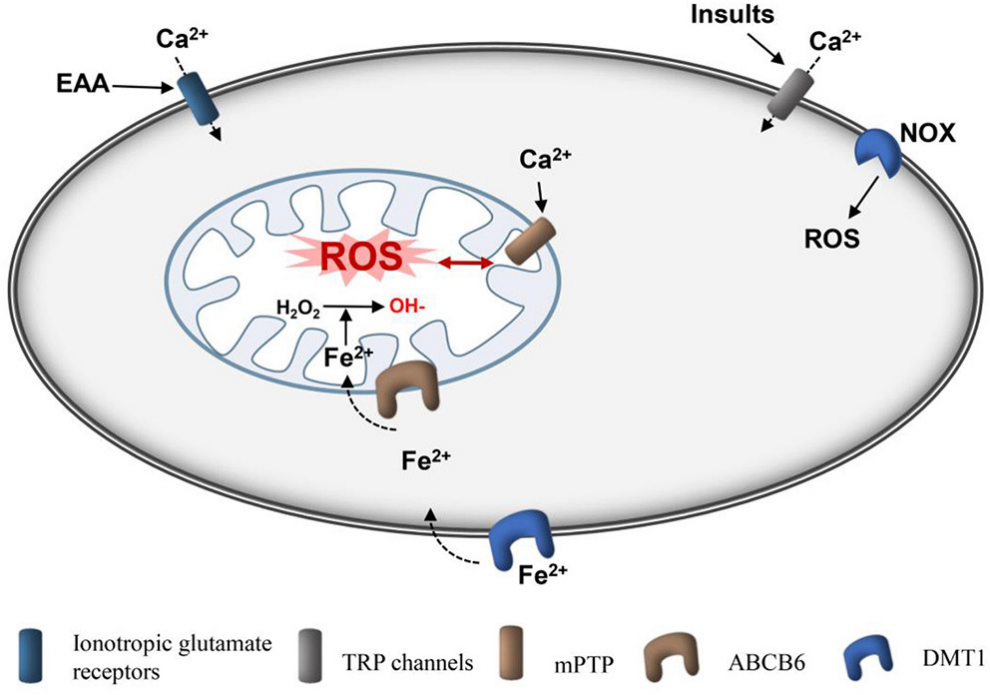
Schematic diagram of production of mitochondrial ROS after ICH. Ionotropic glutamate receptors, such as NMDA receptor and APMA receptor, are activated by glutamate, causing cellular Ca^2+^ overload in cells and mitochondria, respectively. Insults, such as ROS and pressure can induce cellular Ca^2+^ overload directly through TRP channels. Ferrous iron can be transported into cells by DMT1 and then loaded into mitochondria by ABCB6. Ferrous is used to convert H_2_O_2_ to hydroxyl radicals, which are the active radicals causing severe oxidative damage. mPTPs are activated by Ca^2+^ and ROS to open, and ROS are subsequently released. In addition, NOX can produce ROS. ROS, reactive oxygen species; Fe^2+^, ferrous iron; AMPA, amino-3-hydroxy-5-methyl-4-isoxazole-propionic acid receptor; NMDA, N-methyl-D-aspartic acid receptor; fer-1, ferrostatin-1; DMT1, divalent metal transporter 1; mPTP, mitochondrial permeability transition pore; NADPH, nicotinamide adenine dinucleotide phosphate; NOX, nicotinamide adenine dinucleotide phosphate oxidase.

The blood–brain barrier (BBB) is a dynamic interface between the peripheral circulation and the CNS that prevents toxic substances from the CNS and contributes to the maintenance of brain homeostasis (Tschoe et al., 2020). Disruption of the BBB can cause brain edema, which is also an important secondary injury after ICH (Figure 3). Many researchers have demonstrated that mitochondrial membrane potential (MMP)-9 are upregulated after ICH, which is associated with oxidative stress and BBB disruption (Katsu et al., 2010). Inhibition of oxidative stress can decrease MMP-9 levels (Katsu et al., 2010). Therefore, ROS can trigger the activation of MMPs, leading to BBB disruption.

As mitochondria are the main sites of ROS production, mitochondrial accumulation and high oxygen consumption in the CNS render nerve tissue vulnerable to oxidative stress (Mariani et al., 2005; Sidlauskaite et al., 2018; Zheng et al., 2018).

Oligodendrocytes are rich in sphingomyelins, which are prone to injury from oxidative stress, leading to white matter damage (Zhuo et al., 2016). Some non-selective antioxidants, such as edaravone, have been shown to be effective in animal studies but have failed in clinical trials for use in a patient of ICH (Nakamura et al., 2008; Yang et al., 2011, 2015). Recent studies report that selective mitochondrial ROS (mROS) scavengers are superior to non-selective ROS scavengers in the treatment of many REDOX diseases involving mitochondrial dysfunction (Hu et al., 2016; Georgieva et al., 2017; Dey et al., 2018). Our group found that the MitoQ, a mitochondrial reactive oxygen-scavenging agent, can improve mitochondrial function and outcome of mice after ICH (Chen et al., 2020a,b).

Adaptive and non-adaptive responses to REDOX stress may involve mitochondrial channels, such as mitochondrial permeability transition pore (mPTP) and intimal anion channels (IMACs), and activation of these channels leads to an imbalance of REDOX homeostasis in cells and mitochondria, inducing ROS release (Augustynek et al., 2014; Bernardi et al., 2015). The regeneration cycle of the formation and release of mROS is termed as ROS-induced ROS release (Zorov et al., 2014). The mPTP is a multiprotein complex consisting of cyclophilin D (CyPD), mitochondrial peptide proline trans isomerase, voltage-dependent anion channel (VDAC), adenine nucleotide transporter (ANT), and other molecules that form channels in the mitochondrial inner membrane (Li et al., 2014; Bernardi et al., 2015; Chinopoulos, 2018). Regular mPTP opening plays a key physiological role in maintaining a healthy internal mitochondrial environment (Chinopoulos, 2018). At high ROS levels, continuous mPTP opening will trigger ROS release, leading to mitochondrial dysfunction; moreover, the transmission of ROS from damaged mitochondria to neighboring mitochondria results in uncontrollable damage (Zandalinas and Mittler, 2018). In addition, activation of the mPTP, a pore channel in the mitochondrial membrane, may also be a potential mechanism for necrosis and apoptosis (Porter and Beutner, 2018). Overall, mitochondria are the main sites of ROS production and participate in the amplification of ROS (Sies et al., 2017). The increase in mROS that occurs after ICH can be partially reversed by the VDAC inhibitor TRO-19622 or the mROS-specific-scavenging agent Mito-tempo (Ma et al., 2014).

In general, it is vital to explore the protective effect of selective mROS scavengers for treating ICH-induced SBI.

### Mitochondria-Related Neuronal Apoptosis Pathway After ICH

Mitochondria are associated with a variety of apoptotic mechanisms. Apoptosis is a process by which early tissue injury around hematoma occurs after ICH (Lu et al., 2015; Bobinger et al., 2018). There are many factors influencing apoptosis after ICH, such as free-radical cascade reactions, inflammation, cytokines, thrombin, and erythrocyte lysis (Salihu et al., 2016). Apoptosis-related genes are involved in neuronal apoptosis after ICH, including BCL-2 associated x protein (Bax), which promotes apoptosis, and Bcl-2, which inhibits apoptosis (Song et al., 2019; Tang et al., 2020). Bcl-2 and Bax are located in the outer mitochondrial membrane, and the Bcl-2/Bax ratio can reflect the tendency of cells to undergo apoptosis or to survive after stimulation: When the Bcl-2 level increases and the Bax decreases, the ratio increases and cells tended to survive (Chen et al., 2015; Luo et al., 2020). The Bcl-2/Bax ratio in cerebral tissue decreases after ICH, which can be inhibited by melatonin (Wang et al., 2018).

Mitochondria directly participate in the pre-apoptotic signaling pathway by releasing cytochrome C (CytoC) and apoptosis induction factor (AIF), which play a central role in the survival or death of neurons (Sabirzhanov et al., 2016). The latter induces apoptosis by activating caspases, whereas AIF is not related to caspase-mediated apoptosis (Hangen et al., 2010). Changes in mitochondrial outer membrane permeability lead to mitochondrial metabolic failure and the release of caspase-activating molecules as well as caspase-dependent death effectors, inducing mitochondrial swelling and neuron death (Augustynek et al., 2014). Previous studies have reported reduced mitochondrial respiratory function and CytoC release around the hematoma after ICH, suggesting that mitochondrial dysfunction is associated with brain injury (Lu et al., 2015; Ding et al., 2017). Moreover, based on double-labeled immunofluorescence, CytoC mainly colocalizes with CNPase, suggesting that the apoptosis of oligodendrocytes after intracapsular hemorrhage may be mediated by the mitochondrial pathway (Zhuo et al., 2016). CytoC levels reflect whether apoptosis in oligodendrocytes induced by ICH is mediated by the mitochondrial pathway (You et al., 2016). Recent studies have found that the expression of CytoC is upregulated in rats with ICH, which is consistent with the apoptosis trend of oligodendrocytes (Lu et al., 2015). Cell death after ICH can be induced by brain edema, inflammatory reaction, and BBB disruption, which are the results of secondary injury.

### Mitochondrial Dynamics and ICH

**Figure 4:**
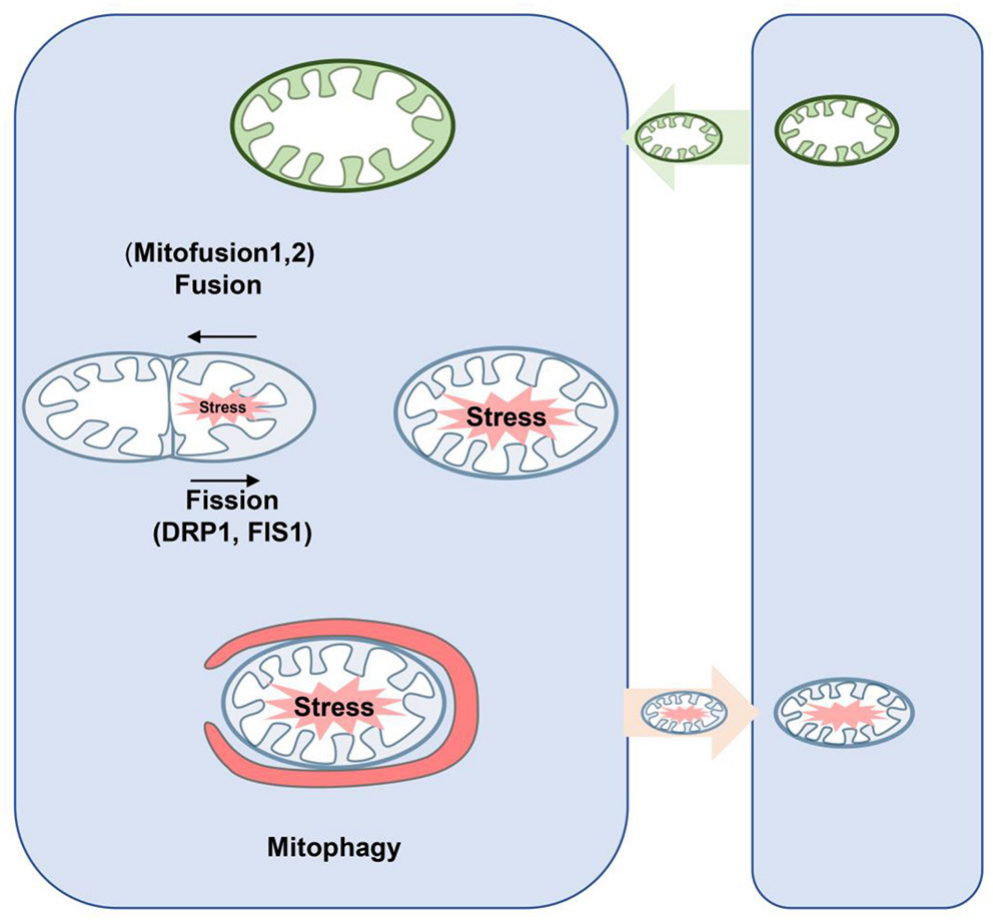
Schematic diagram of mitochondrial dynamics and mitophagy. Fusion is regulated by Mfn1/2 and Opa1, which mediate fusion of the outer and inner mitochondrial membrane, respectively. In the process of fission, Drp1 is recruited from the cytoplasm to the outer membrane of mitochondria and interacts with its receptor protein and Fis1 to form the fission complex in the outer membrane. Thus, damaged mitochondria can be repaired through fusion with healthy mitochondria; mitochondrial fission allows damaged mitochondria to be separated and eliminated by mitophagy. Neurons may secrete mitochondria, and these mitochondria may be unhealthy but may accept healthy mitochondria at the same time to maintain normal neuronal function. Drp, dynamin-related proteins; Mfn1/2, mitofusins; Opa1, optic atrophy 1.

Abnormal regulation of mitochondrial dynamics, which shifts the balance of fusion and fission to fission, is involved in the pathological process of hemorrhagic SBI (Wu et al., 2020a). Mitochondria are highly dynamic organelles that constantly undergo fission and fusion to maintain normal morphology and function (Fenton et al., 2020), as characterized by highly coordinated fission (the separation of a single mitochondria into two or more daughter organelles), fusion (an opposing reaction), and transport to a specific location in the cell (Giacomello et al., 2020) (Figure 4). These dynamic processes are accompanied by changes in mitochondrion size and shape (Pekkurnaz et al., 2014).

Under physiological conditions, mitochondrial fission is crucial for removing damaged mitochondria to maintain mitochondrial stability (Weir et al., 2017). However, excessive fission damages mitochondrial structure, leading to impaired respiratory function, increased mROS production, ATP deficiency, and apoptotic pathway activation (Nakamura and Lipton, 2011; Zhou et al., 2019; Rong et al., 2020).

In turn, mitochondrial division is mainly regulated by dynamic protein-related protein 1 (Drp1), a membrane protein, which is recruited into the mitochondrial outer membrane and triggers mitochondrial division (Montessuit et al., 2010). Phosphorylation is a key step in regulating Drp1 recruitment under different conditions (Kashatus et al., 2011). Phosphorylation at Ser616 (S616) is generally believed to accelerate its recruitment to the mitochondrial membrane, whereas phosphorylation at S637 inhibits this process (Xu et al., 2016; Ma et al., 2020). Mitochondrial quality control mediated by Drp1 and mfn1/2 has been shown to play a crucial role in preventing ICH injury, such as neural apoptosis, brain edema, and inflammatory response (Wu et al., 2020a,b).

Previous studies have shown that the Drp1 inhibitor mdivi-1 can attenuate oxidative stress and neuronal apoptosis after subarachnoid hemorrhage (Fan et al., 2017). Furthermore, recent studies have shown that Drp1 inhibitors exert neuroprotective effects in the ICH models (Wu et al., 2020a,b), suggesting that excessive Drp1 activity may be an important risk factor for ICH-induced brain injury. In fact, mitochondrial injury induced by acrolein around the hematoma after ICH is associated with increased Drp1 translocation and excessive mitochondrial fission, and acrolein-scavenging agents can significantly inhibit Drp1-mediated nuclear fission after ICH and reduce mitochondrial morphological damage (Wu et al., 2020a). Most importantly, inhibition of mitochondrial fission significantly alleviates neuronal apoptosis, cerebral edema, and neurological deficits after ICH (Wu et al., 2020c).

### Mitophagy and Its Role in ICH

Mitophagy was first described by Lemasters (2005), who determined that mitophagy is an autophagic response that is responsible for the specific removal of damaged mitochondria to maintain mitochondrial homeostasis and is an important mechanism for maintaining mitochondrial health (Lemasters, 2014) (Figure 4). Recently, it has been found that the inhibition of mitophagy is beneficial in the pathogenesis of various neurological diseases, such as subarachnoid hemorrhage (Li et al., 2014; Zhang et al., 2019). Mechanically, PINK1/Parkin-mediated fission is the most characteristic mitophagy pathway (Geisler et al., 2010). Moreover, other E3 ubiquitin-protein ligases, such as SMURF1, SIAH1, MUL1, Gp78, ARIH1, and HUWE1, are involved in the regulation of PINK1-Parkin independent mitophagy (Li et al., 2014).

In various pathological environments, “alternative” activation pathways of mitophagy are also involved in mitophagy (Ma et al., 2019). As an example, it has been demonstrated that FUNDC1, an OMM protein, is able to activate mitophagy. Indeed, FUNDC1 can directly recruit LC3 through its LIR domain [36]. Bcl-2-like 13 (BCL2 L13), another OMM protein and a homolog of Atg32, is a mitochondrial autophagy receptor in yeast that can also directly bind to LC3 and promote mitochondrial autophagy through its LIR motif (Jing et al., 2012). Recently, it has been found that mitophagy is involved in the pathogenesis of various neurological diseases, such as subarachnoid hemorrhage (Li et al., 2014; Zhang et al., 2019).

In general, it appears that the effect of mitophagy depends on its severity. Under physiological conditions, mitophagy prevents accelerated cellular senescence and programmed cell death (Kang, 2020; Zhou and Tan, 2020). However, excessive mitophagy plays a lethal role when overactivated by severe pathological stress like ischemia (Huang et al., 2020). NIX primarily regulates the basal level of mitophagy in physiological conditions, whereas BNIP3 exclusively activates excessive mitophagy, leading to cell death (Shi et al., 2014), although the role of mitophagy in ICH has not been fully appreciated to date. Further research is needed to understand whether mitophagy is beneficial after ICH.

### Mitochondrial Membrane Potential

In cells, the MMP is typically maintained between 80 and 140 mV (Cloonan et al., 2016) and is regulated by mPTP. Although the structure and composition of mPTP remain controversial, CyPD has long been recognized as a pivotal regulator of the pore openness (Baines and Gutierrez-Aguilar, 2018; Porter and Beutner, 2018). The activity of CyPD is mainly regulated by post-translational modifications, including acetylation, S-glutathione, glycosylation, and S-nitrification (Hafner et al., 2010).

The mitochondrial injury induced by ICH is the result of mPTP opening, whereby various proteins, such as CytoC, are released into the cytoplasm, constituting important events that lead to apoptosis (Tomasello et al., 2009; Bock and Tait, 2020). Melatonin inhibits mPTP opening by upregulating antioxidants to reduce mitochondrial dysfunction therefore inhibiting SBI after ICH (Wang et al., 2018). It has been shown that melatonin treatment significantly inhibits apoptosis and mitochondrial damage, findings verified *in vivo* and *in vitro*, and some researchers have found that melatonin can significantly inhibit apoptosis and mitochondrial damage *in vivo* and *in vitro* (Wang et al., 2018). Our group found that selective mROS antioxidants MitoQ, but not the non-selective antioxidants, almost completely attenuated the iron-induced membrane potential decrease and cell death (Chen et al., 2020b). Additionally, Honokiol (HKL, a pharmacological agonist of sirt3) protects against hyperglycemic ICH-induced neuronal injury *via* the inhibition of mitochondrial depolarization (Zheng et al., 2018).

In addition, Miro1 is a glutamate receptor-dependent calcium sensor that targets the mitochondria of neurons, connecting mitochondria with motor proteins (Macaskill et al., 2009). The Miro1 protein is rapidly ubiquitinated and exhausted in damaged mitochondria to block microtubule-dependent transport of damaged mitochondria, thus promoting mitophagy in mitochondria with decreased membrane potential (Hsieh et al., 2019). Chen et al. reported that the upregulation of Miro1 significantly alleviated pathological symptoms on SBI *in vivo* and *in vitro* (Li et al., 2020). Miro1 may also be involved in a key mechanism for mitochondrial quality control after ICH.

### Mitochondrial Transfer

Hayakawa et al. (2016) demonstrated for the first time in 2016 that astrocytes can transfer functional mitochondria to neighboring neurons and promote the survival of receptor neurons (Figure 4). These transferred mitochondria are involved in many important functions that benefit the recipient cells. In addition, these groups suggested that mitochondrial transfer improves functional neuron damage after stroke (Hayakawa et al., 2016).

Astrocytic release of extracellular mitochondria particles was mediated by a calcium-dependent mechanism involving CD38/cyclic ADP ribose signaling. Transient focal cerebral ischemia in mice induced astrocytic mitochondria entry to adjacent neurons that amplified cell survival signals (Hayakawa et al., 2016). In addition, damaged cells can produce phosphatidylserine, inducing the tunneling nanotubes (TNTs) formation promoting mitochondrial transfer (Liu et al., 2014). Similarly, TNT mitochondrial transfer from mesenchymal stem cells to cardiomyocytes improved survival and reduced cellular damage in an *in vitro* ischemia/reperfusion model (Han et al., 2016).

Studies have shown that the main function of transferred mitochondria is to enhance the capacity of recipient cells to metabolize energy (Picca et al., 2019). It has also been reported that mitochondria-derived humanin (HN), a cross-cellular signaling molecule, promotes neuron recovery after ICH: Mitochondrial transfer and the microglia phenotype regulate the function of “reparative” microglia after astrocytes are secreted as peptides or transferred among mitochondria (Chou et al., 2017). Additionally, neurons may secrete mitochondria, but these mitochondria may be unhealthy and may be considered cellular waste (Chou et al., 2017).

## Perspectives and Conclusions

Mitochondrial dysfunction is an early initiating event in the pathophysiology of ICH. Bioenergy genetic defects, structural abnormalities and mitochondrial morphology, and aberrant mitochondrial dynamics play an important role in the activation of neuronal death signaling pathways. In preclinical studies, interventions aimed at mitochondrial quality control and mitochondrial dynamics have been shown to have neuroprotective effects (Chimeh et al., 2018).

Recent evidence of mitochondrial transfer has provided a new perspective for intercellular communication. The latest research suggests that mitochondria themselves can act as “help me” signals to respond to different extracellular stimuli and recruit adjacent cells to rescue those injured in stroke and those affected by aging, and disease (Heyck et al., 2019; Gollihue and Norris, 2020). Clearance of damaged mitochondria and replacement with healthy organelles is another promising treatment for CNS diseases, where mitochondria are abundant in distal axonal synapses and dendrites (He et al., 2020). The detailed mechanisms of mitochondrial release and receptor recognition in donor cells, the generalizability of beneficial results, and the ethical implications associated with the artificial transfer of mitochondria remain to be further studied.

Here, Table 1 lists the potential translational and clinical strategies targeting mitochondria in CNS disease. We have listed mitochondrial protectants that were used to protect neuronal injury from diseases occurring due to damage in the nervous system. Some of them, such as edaravone, are effective to improve prognosis in the studies of ischemic stroke (Shinohara et al., 2009). However, Cyclosporine was generally not effective in reducing infarct size after stroke (Nighoghossian et al., 2015). Regrettably, these drugs have not yet been tested in ICH.

**Table 1:** Registered drugs targeting mitochondrial dysfunction on ClinicalTrials.gov.

**Condition/Disease**	**Status**	**Phase**	**Treatment**	**Trial number**
Parkinson's disease	Completed	2	Mitoquinone	NCT00329056
Stroke	Completed	2	Cyclosporin A	NCT01527240
Glucose metabolism disorders	Active, not recruiting	Not applicable	NMN	NCT03151239
Progressive supranuclear palsy Neurological disorders	Completed	2 and 3	CoQ_10_	NCT00532571
Sleep disorder Traumatic brain injury	Withdrawn	2	Tesamorelin	NCT02931474
Mild cognitive impairment	Recruiting	1	Niagen®	NCT03482167
Friedreich's ataxia	Completed	3	Idebenone	NCT01303406
Brain tumor treated with cranial or cranial-spinal radiation	Completed	3	Metformin	NCT02040376
Cerebral infarction	Completed	4	Edaravone	NCT00200356

The clinical trials using mitochondrial protectants or antioxidants have not been successful or fulfilled yet. ROS and mitochondrial damage are produced rapidly and have a cascade amplification effect (Qu et al., 2016). This time window of administration may be a problem for patients. In future clinical research, researchers should pay attention to the time window of antioxidant drugs to improve the therapeutic effect of ICH. In addition, a more comprehensive study of the mechanism of mitochondrial damage can also provide a more perfect strategy for the treatment of ICH.

## Author Contributions

WC, CG, and YC drafted the manuscript and figures. HF and YC proofread and revised the manuscript. YC gave the final proof for this submission. All authors contributed to the article and approved the submitted version.

## Conflict of Interest

The authors declare that the research was conducted in the absence of any commercial or financial relationships that could be construed as a potential conflict of interest.
